# Invasive Coronary Angiography after Chest Pain Presentations to Emergency Departments

**DOI:** 10.3390/ijerph17249502

**Published:** 2020-12-18

**Authors:** Frank M. Sanfilippo, Graham S. Hillis, Jamie M. Rankin, Donald Latchem, Carl J. Schultz, Jongsay Yong, Ian W. Li, Tom G. Briffa

**Affiliations:** 1 School of Population and Global Health, The University of Western Australia, Perth 6009, Australia; ian.li@uwa.edu.au (I.W.L.); tom.briffa@uwa.edu.au (T.G.B.); 2 Cardiology Department, Royal Perth Hospital, Perth 6000, Australia; graham.hillis@uwa.edu.au (G.S.H.); carl.schultz@uwa.edu.au (C.J.S.); 3 Medical School, The University of Western Australia, Perth 6009, Australia; 4 Cardiology Department, Fiona Stanley Hospital, Murdoch 6150, Australia; james.rankin@health.wa.gov.au; 5 Department of Cardiovascular Medicine, Sir Charles Gairdner Hospital, Nedlands 6009, Australia; donald.latchem@health.wa.gov.au; 6 Melbourne Institute of Applied Economic and Social Research, University of Melbourne, Melbourne 3010, Australia; jongsay@unimelb.edu.au

**Keywords:** chest pain, emergency departments, coronary angiography, linked health data, cardiac biomarkers, health services research, gender differences

## Abstract

We investigated patients presenting to emergency departments (EDs) with chest pain to identify factors that influence the use of invasive coronary angiography (ICA). Using linked ED, hospitalisations, death and cardiac biomarker data, we identified people aged 20 years and over who presented with chest pain to tertiary public hospital EDs in Western Australia from 1 January 2016 to 31 March 2017 (ED chest pain cohort). We report patient characteristics, ED discharge diagnosis, pathways to ICA, ICA within 90 days, troponin test results, and gender differences. Associations were examined with the Pearson Chi-squared test and multivariate logistic regression. There were 16,974 people in the ED chest pain cohort, with a mean age of 55.6 years and 50.7% males, accounting for 20,131 ED presentations. Acute coronary syndrome was the ED discharge diagnosis in 10.4% of presentations. ED pathways were: discharged home (57.5%); hospitalisation (41.7%); interhospital transfer (0.4%); and died in ED (0.03%)/inpatients (0.3%). There were 1546 (9.1%) ICAs performed within 90 days of the first ED chest pain visit, of which 59 visits (3.8%) had no troponin tests and 565 visits (36.6%) had normal troponin. ICAs were performed in more men than women (12.3% vs. 6.1%, *p* < 0.0001; adjusted OR 1.89, 95% CI 1.65, 2.18), and mostly within 7 days. Equal numbers of males and females present with chest pain to tertiary hospital EDs, but men are twice as likely to get ICA. Over one-third of ICAs occur in those with normal troponin levels, indicating that further investigation is required to determine risk profile, outcomes and cost effectiveness.

## 1. Introduction

Chest pain is one of the most common presenting conditions to emergency departments (EDs) worldwide, with over 6.5 million presentations per year in the USA [1]. In Australia, there are over 500,000 ED visits per year with a presenting complaint of chest pain [2]. This has resulted in ‘pain in throat and chest’ being the 2nd most common principal diagnosis on ED discharge, accounting for over 300,000 discharges and increasing each year [3]. Invasive coronary angiography (ICA) is the standard and well-established diagnostic method for detecting coronary artery disease in patients presenting with chest pain of suspected cardiac origin. It is recommended in clinical guidelines as the initial step in deciding whether coronary artery revascularisation procedures are required in the management of acute coronary syndromes (ACS) [4]. However, the majority of ED presentations for chest pain do not result in a diagnosis of ACS or other coronary heart disease [5].

The risk of ACS in patients presenting with chest pain is assessed through symptoms and cardiovascular risk factors, serial electrocardiographs (ECGs) and biomarkers of myocardial injury [4]. In particular, the uptake of high-sensitivity troponin in EDs in recent years has allowed more rapid evaluation of chest pain presentations [6]. The overall cost of investigating and managing coronary heart disease in Australian public hospital EDs was AU$147 million in the period 2015–2016, with additional expenditure of AU$1.5 billion for private hospital services and public hospital admissions [7]. Despite this financial burden, little is known about patient profiles and the use of high-cost investigations such as ICA in the population of patients presenting with chest pain to EDs.

The aim of our study was to describe the patient characteristics, ED discharge diagnosis, determinants and use of ICA following ED presentations for chest pain in Western Australia (WA) in the period 2016–2017. Our motivation was to understand patient profiles and the scenarios by which patients are admitted for ICA given the increasing ED presentations for chest pain in Australia [3], and increasing use of ICA [8]. This will inform current clinical practice and health policy.

## 2. Materials and Methods

### 2.1. Data Source and Study Cohort

Study datasets of linked administrative records were obtained from the Western Australian Department of Health consisting of hospital morbidity data (inpatient admissions), ED visits and death registrations. These are core datasets of the Western Australian Data Linkage System in which records belonging to the same person are linked using a dynamic probabilistic matching algorithm [9]. Our study cohort (‘ED chest pain cohort’) consisted of people aged 20 years or older who presented with chest pain in EDs of adult tertiary public hospitals in WA from 1 January 2016 to 31 March 2017. Patients were identified from the linked ED visits dataset by searching for “chest pain” and variations (including spelling mistakes) in the presenting complaint text field, or specific codes in the symptom field.

The ED chest pain cohort was also linked to troponin results from Pathwest Laboratory Medicine that provides pathology services to the tertiary hospitals. Our ED dataset did not have the ED discharge date and time. Hence, linkage of troponin data was based on the period from (ED presentation time—1 h) to (ED presentation time + 6 h) to capture all cardiac biomarker tests where blood samples were collected within 1 h prior to the ED admission time and up to 6 h after the ED admission time. Troponin test results were classified as normal or elevated based on the gender-specific assay threshold for the laboratory, then further classified as all tests normal, all tests elevated or at least one test elevated with results showing a rising or falling pattern [10]. The troponin test results were considered elevated if they satisfied either of the two latter conditions.

Study approval was received from the Western Australian Department of Health Human Research Ethics Committee and Research Governance Office (RGS 2876), and the Human Research Ethics Committee of The University of WA (RA/4/1/7230).

### 2.2. Emergency Department Discharge Pathways

Patients in the ED chest pain cohort were grouped into the following ED discharge categories based on ICD-10-AM [11] codes from their ED discharge diagnosis in the ED dataset: (i) chest pain (R07.1-R07.4); (ii) acute myocardial infarction (MI, I21); (iii) unstable angina (I20.0); (iv) other angina (I20.1-I20.9); (v) other coronary heart disease (I22-I25); (vi) other cardiovascular disease (I00-I15.9, I26-I99); and (v) non-cardiovascular disease (all other ICD-10-AM codes). Using hospital admissions, ED and death study datasets, we identified 5 possible pathways following discharge from ED: (1) discharged home direct from ED; (2) died in hospital after inpatient admission; (3) admitted as a hospital inpatient and survived; (4) transferred to another tertiary hospital ED then admitted, discharged home or died; and (5) died in ED during the initial visit. Patients who survived the ED visit may have subsequently died and we report all-cause death within 30 and 90 days from the ED chest pain visit.

### 2.3. Invasive Coronary Angiography

Coronary angiograms can be performed following any of the ED discharge pathways (except if died in ED), including repeat angiograms after an initial procedure. We used the linked hospital admissions data to identify ICAs from any of the procedure fields within 90 days following ED discharge pathways 1, 3 and 4. This included the initial and any repeat angiogram procedures within 90 days of a visit to the ED for chest pain. If hospital admission records following the ED presentation had a coronary artery revascularisation procedure (percutaneous coronary intervention or coronary artery bypass graft surgery) coded, but no procedure codes for coronary angiography, then we assumed that angiography was performed and not coded. In this case, the assumed angiography was counted together with angiogram procedures that were coded in the hospital admissions data. This allowed a more complete identification of ICA following the ED presentation. Coronary computed tomography angiography (CCTA) is a non-invasive modality with an emerging role in the investigation of chest pain as an alternative to ICA, especially in low-risk patients [12,13]. However, in WA, it is mainly used in the private sector or public hospital outpatients.

### 2.4. Statistical Analysis

A flowchart was constructed showing the five ED discharge pathways for the ED chest pain cohort, with counts and proportions representing the number of visits to ED or hospital inpatient admission. Frequencies were reported for the number of ICAs and the ED discharge diagnosis of the cohort. For visits that resulted in an inpatient admission (pathways 2 and 3), a bivariate analysis of ED discharge diagnosis and principal discharge diagnosis of the corresponding inpatient admission revealed how the diagnoses changed from ED to inpatient. We used the 2-sided Pearson Chi-squared statistic or Fisher’s exact test to test for associations between troponin test results, sex, age group, and remoteness area and use of ICA within 90 days of the ED presentation. Differences between men and women were compared for age, remoteness area, ED discharge diagnosis, 10-year comorbidity history, troponin test results and initiation of ICA. These were assessed from the first ED presentation for chest pain for each person in the ED chest pain cohort for a person-based analysis. A multivariate logistic regression model was used to further investigate any gender differences by estimating the odds of initiating ICA in men compared with women (as reference level) adjusting for age (continuous variable), remoteness area, ED discharge diagnosis and troponin test results.

Additional stratified analyses assessed the use of angiograms within 90 days from the first ED chest pain presentation compared with use beyond 90 days (to 30 June 2017). Residential remoteness was assessed by remoteness areas which divide Australia into 5 classes of remoteness based on the Accessibility and Remoteness Index of Australia (ARIA+) using Statistical Area Level 2 (SA2) from the 2011 census (2016 SA2 data were not yet available) [14]. For the analysis we grouped this as major cities, inner/outer regional, remote/very remote and missing. History of comorbidities and coronary artery revascularisation procedures were identified from linked records in the hospital admissions dataset with a 10-year lookback from the ED presentation date. Coronary artery revascularisation procedures were also identified from the hospital admissions dataset for up to 90 days from the ED presentation date.

## 3. Results

### 3.1. Cohort Characteristics

Characteristics of the ED chest pain cohort are shown in Table 1. During the study period, there were 20,131 presentations to Western Australian tertiary public hospital EDs for chest pain from 16,974 people, with 4.8% Indigenous and the majority (92.9%) residing in major cities (population ≥ 250,000) in WA. Males (50.7%) and females were equally represented with a mean age of 55.6 years (range 20–103 years). Younger age groups (<65 years) were more common than those 65 years and older (65.4% vs. 34.7%). There were 149 (0.9%) deaths within 30 days of the first ED chest pain presentation in the cohort, and 311 (1.8%) deaths within 90 days. There were 1958 (11.5%) people with two or more ED chest pain visits, and this subgroup was older with a higher prevalence of comorbidities (Table 1).

### 3.2. ED Discharge Diagnosis

**Figure 1:**
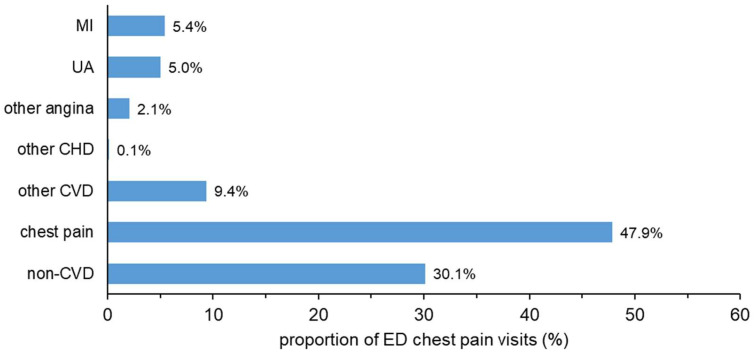
ED discharge diagnosis as a proportion of total number of visits for the ED chest pain cohort (*n* = 20,131). ED, emergency department; MI, myocardial infarction; UA, unstable angina; CHD, coronary heart disease; CVD cardiovascular disease.

**Figure 2:**
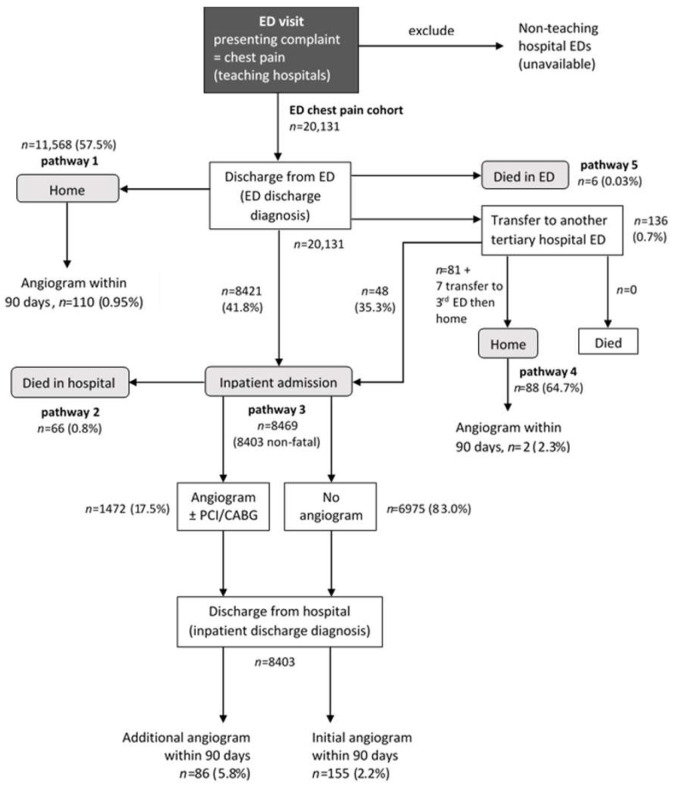
Flowchart of the five pathways following chest pain presentation in the emergency department (ED) for the ED chest pain cohort, with some leading to invasive coronary angiography. Counts represent number of visits. PCI, percutaneous coronary intervention; CABG, coronary artery bypass graft surgery.

The majority of ED visits for chest pain resulted in an ED discharge diagnosis of chest pain (47.9%, Figure 1), with non-cardiovascular diagnoses being the next most common (30%). Cardiovascular diagnoses were less common, with 10.4% of chest pain visits resulting in an ED discharge diagnosis of ACS (Figure 1). Supplementary Table S1 compares the ED discharge diagnosis with the corresponding principal discharge diagnosis of the inpatient admission for patients admitted to hospital directly from the ED (pathways 2 and 3 from Figure 2). There was a large difference between the ED discharge diagnosis and the inpatient discharge diagnosis, except for MI (68.5% of ED visits resulted in the same diagnosis), chest pain (57% the same) and non-coronary heart disease diagnoses (85% the same).

### 3.3. ED Discharge Pathways

Figure 2 shows and quantifies the five pathways identified following discharge from ED after the chest pain presentations. The dominant pathways (1 and 3) were discharge to home directly from ED (*n* = 11,568 visits, 57.5%) and non-fatal hospital inpatient admission (*n* = 8403, 41.7%) respectively. Together, these make up 99.2% of the ED discharge pathways in the ED chest pain cohort. A small proportion of the cohort died following inpatient admission from ED (pathway 2, *n* = 66, 0.3%) or in ED during the initial visit (pathway 5, *n* = 6, 0.03%). Pathway 4 was transfer to another tertiary hospital ED then discharge home without inpatient admission (*n* = 88, 0.4%). Pathways 1, 3 and 4 led to ICA (Figure 2). In visits where the patient was discharged home from ED (pathway 1), only 1.3% had an ICA within 90 days (booked or emergency). Following inpatient admission (pathway 3), angiograms within 90 days were either done during the admission (17% of non-fatal admissions) or after inpatient discharge.

### 3.4. Troponin Tests and Coronary Angiography

High-sensitivity troponin I was the exclusive cardiac biomarker test completed in the ED chest pain cohort (Supplementary Table S2). However, 27.8% of the ED chest pain visits did not have any troponin tests recorded in the pathology dataset, whilst 70.9% had 1 or 2 tests. In total, 72.2% (*n*= 14,542) of ED visits had a troponin I test(s) completed. Patients who had an ED discharge diagnosis of MI or other coronary heart disease were those with the highest proportion of elevated troponin I test results (Supplementary Table S3). Patients with unstable angina or other angina as an ED discharge diagnosis were more likely to have normal troponin results. Patients with an ED discharge diagnosis of chest pain, non-cardiovascular disease or other cardiovascular disease were more likely to have no troponin tests done or to have normal test results. Test results were below the gender-specific upper limit of normal for all troponin tests in 81% of the 14,542 ED visits where troponin tests were completed.

Patients with elevated troponin test results were more likely to have the initial ICA within 90 days of their first ED chest pain visit (Supplementary Table S4). Table 2 shows 1546 admissions (9.1% of patients in the ED chest pain cohort) for initial ICA that were done within 90 days of the first ED presentation for chest pain. There was a significant association between troponin I test results and timing of the initial angiogram (*p* < 0.0001). There were 59 (3.8%) initial angiograms in which no troponin test results were recorded. Patients with elevated troponin I test results were more likely to have an initial angiogram within 7 days of the ED presentation (94% completed in the first 7 days). There were 565 (36.6%) initial angiograms completed in patients who had all troponin test results normal. Patients who had angiograms following normal troponin levels had a high prevalence of coronary heart disease (46.9%, Supplementary File Table S5).

Coronary artery revascularisation procedures were identified in 1064 (6.3%) patients following the ED presentation, with 925 (86.9%) of these having the procedure within 90 days of the ED presentation. There was a significant association between time to first revascularisation procedure and troponin test results. Of the 1064 patients, revascularisation procedures within 90 days were done in 25 (68%) of those with no troponin tests, 266 patients (74%) who had normal troponin tests and 634 (95%) who had at least one elevated troponin result (*p* < 0.0001).

### 3.5. Gender Differences and Other Determinants of Angiography

There was a significant difference in the proportion of angiograms initiated in men and women, with twice as many men having initial angiograms within 90 days of the first ED chest pain visit (12.3% vs 6.1%, *p* < 0.0001, Supplementary Table S6). This is likely due to differences between males and females in age, remoteness area, ED discharge diagnosis, comorbidity history and troponin test results (Supplementary Table S6). The gender difference remained after adjusting for these variables in a logistic regression model (adjusted odds ratio 1.89, 95% CI 1.65, 2.18; C statistic 0.89; Table 3). However, there were no differences in the timing of coronary angiography between men and women following the first ED chest pain presentation, with 84% of procedures initiated in the first 7 days in both men and women, and proportions decreasing out to 90 days (*p* = 0.28, Table 2). There were no significant differences in the timing of initial angiography by age group (*p* = 0.09) or remoteness area (*p* = 0.45) within 90 days of the first ED chest pain presentation (Table 2). However, a higher proportion of patients from remote and very remote areas had their angiography procedure within 7 days of the ED visit (91% vs 84% for major cities).

The odds of initiating ICA within 90 days are shown in Table 3 for various patient demographic and clinical characteristics. The odds were 2.1 to 2.5 times higher in people from regional, remote and very remote areas than those from city areas. However, the strongest effects were from the ED discharge diagnosis (particularly MI, unstable angina or other CHD), as well as troponin level. The model variables are indicators of how clinicians would be thinking when assessing the need for ICA. They suggest that the ED discharge diagnosis is the single most important factor, followed by an elevated troponin level, that determines the likelihood of ICA within 90 days of the ED chest pain visit. The very high C statistic of 0.89 for the model suggests that there are very few other factors that would be considered in the decision to initiate ICA or not.

## 4. Discussion

In this study, we investigated the use of ICA in patients who present with chest pain to tertiary public hospital EDs and the different pathways by which this occurs. We found that men and women are equally represented in the group of people who visit EDs for investigation of chest pain, but have different demographic and clinical characteristics. Overall, 9% of the cohort had ICA within 90 days of the ED chest pain presentation, with men twice as likely as women (12% vs. 6%). However, there was no difference in the timing of the coronary angiogram, with 84% of procedures initiated in the first 7 days in both men and women. Patients in the cohort who had two or more ED chest pain presentations had some differences in characteristics to the total cohort, reflected as older age and a higher prevalence of comorbidities. This subgroup would require further study and assessment of the costs associated with their additional use of healthcare resources.

Approximately half of the chest pain visits had an ED discharge diagnosis of non-specific chest pain, and a further 30% were diagnosed with non-cardiovascular conditions. Hence, only approximately 20% of chest pain visits had a cardiovascular diagnosis on discharge from ED. ACS as an ED discharge diagnosis was identified in 10.4% of presentations to the EDs for chest pain (12.3% in men and 7.6% in women). Whilst this important measure is not reported in Australian national statistics on ED care, Cullen et al. found a similar proportion of 11% for ACS as an ED discharge diagnosis in their single-centre study of ED chest pain presentations in Australia from 2008 to 2011 [5]. In the USA, the proportion of ED chest pain visits that had an ED diagnosis of ACS decreased from 23.6% in 1999–2000 to 13.0% in 2007–2008 [15]. Our study fills the void on the use of ICA in all patients presenting to ED with chest pain, importantly stratified by troponin test results. Over one-third of ICAs are performed in patients with all troponin levels normal, and of these, 70% are in the first week. Further, for the first time, we report on the discrepancy between diagnoses at discharge from ED compared with the final diagnosis in the vast majority admitted to hospital. We have also described the specific cardiovascular diagnoses identified at discharge from ED, which are not routinely presented in reports of national statistics. Furthermore, we have reported the determinants for initiating ICA within 90 days which showed that ED discharge diagnosis and elevated troponin level are the most important factors in the clinical decision for ICA.

Pathways to coronary angiography mostly involved discharge home from ED with subsequent admission for angiography or direct admission to hospital inpatients with/without immediate angiography and discharge home with/without subsequent admission for initial or additional coronary angiography. This reflects the patient casemix where the lower-risk patients (e.g., non-ACS) may not require immediate ICA, whilst the smaller group of patients with an ED diagnosis of ACS would require more urgent diagnostic and therapeutic attention. There also appeared to be consideration of specific patient circumstances. For example, ICA in patients residing in remote or very remote locations were more likely to be done within 7 days of the ED chest pain visit than for patients living in major cities. This may reflect a patient convenience and elucidation of the presenting complaint by initiating the procedure whilst they are in the tertiary hospital rather than discharging them home with a subsequent admission for an elective angiography (pathway 1). This may be more clinically and cost effective in large jurisdictions as in Australia where ICA services are located mostly in major cities, with patients from rural and remote areas having to travel long distances to receive these procedures.

Whilst the majority of ICAs performed was in patients with elevated troponin levels, 37% were completed in patients with normal troponin levels. Almost half of these patients had a history of coronary heart disease, and angiography may have been initiated for evidence of disease progression despite the normal troponin. The clinical and cost implications of this requires further investigation. In addition, 27.8% of chest pain visits did not have any troponin tests recorded in the linked pathology data. The majority of these patients had a non-coronary heart disease diagnosis on ED discharge. However, 4% (*n* = 59) of ICA within 90 days of the ED visit were from visits with no troponin tests. Approximately half of the patients (*n* = 25) with no troponin tests had a coronary artery revascularisation procedure within 90 days of the ED visit. This may partially explain the use of ICA in those with no troponin tests, but we were not able to determine other reasons in this group of patients. Whilst high-sensitivity troponin was thought to aid in the precision of diagnosing ACS [16], its real benefit is in identifying people at low risk of death or MI presenting to ED with chest pain [17].

Heterogeneity in patient characteristics may explain the observed gender differences in the use of ICA, with twice as many men having an angiogram within 90 days as women (adjusted odds ratio 1.89, 95% CI 1.65, 2.18). The variation was only in frequency rather than timing of the procedure following the ED chest pain visit. Men were significantly younger, were less often from major cities, were more likely to have an ED discharge diagnosis of ACS, had a higher prevalence of hospitalised comorbidities (coronary heart disease, diabetes, hypertension and atrial fibrillation) and were more likely to have troponin tests and elevated troponin test results. These observations indicate that they may have been considered higher risk than women, and future analyses of outcomes of the ED chest pain cohort will investigate this. Variations in coronary angiogram rates have been observed in Australia, but not related to clinical need although there is a modest positive correlation between ICA rates and ACS rates [18]. This may also reflect the well-known gender differences in ACS with women having different symptom profiles, longer times from symptom onset to ED presentation, and coronary and non-coronary biological differences [19].

### Strengths and Limitations

The strength of our study was the use of contemporary population-based linked administrative data covering ED presentations, hospital inpatient admissions and death. This allowed identification of chest pain presentations at multiple tertiary ED sites with complete follow up of patients to determine frequency and timing of ICA in any coronary angiography capable sites. There was also complete capture of all-cause death, both in-hospital and out-of-hospital deaths. The hospital inpatient admissions data covered both private and public hospitals throughout the state, so all admissions to hospital following the ED presentation would have been captured. Linkage to cardiac biomarker data allowed an additional level of interpretation of findings based on troponin testing which is part of the diagnostic pathway in managing presentations to ED for chest pain.

The ED data did not include discharge date and time, so biomarker records from the hospital inpatient admission may have incorrectly linked to the ED visit. To account for this, we limited the linkage window to 1 h before and 6 h after the ED presentation time to minimise the number of hospital biomarker tests that would have linked to the ED visit. Hence, ED visits longer than 6 h would not have linked any biomarker tests from blood collected after 6 h in ED. However, this would have minimally affected the number of high-sensitivity troponin tests that were associated with the ED visit because serial testing of high-sensitivity troponin is separated by 2–3 h [4,16]. It is also possible that some people had recent troponin tests prior to their ED visit, and this may have been a reason for not ordering further tests in ED. Given the specialized use of high-sensitivity troponin testing, its time dependence and its importance in diagnosing presentations of chest pain, we expect that any tests completed outside of the teaching hospital EDs would not have impacted significantly on the requirement for testing in the EDs.

We did not have any ECG data or symptoms for the ED chest pain presentations, and relied on the diagnosis made by attending clinicians at the time of the presentation in each tertiary site. This was recorded in the administrative ED dataset from the clinical diagnosis made by doctors based on all diagnostic information available during the ED visit. Tertiary EDs have standard clinical guidelines on the management and diagnosis of chest pain, ACS and other coronary heart disease and this is based on high-sensitivity troponin, not just ECGs. The ECG is used to identify the presence of ST elevation which determines the type of initial treatment required (e.g., primary percutaneous coronary intervention if there is ST elevation MI). In this study, we were interested in diagnostic ICA rather than treatment, and so the troponin tests were of greater importance. Some patients had coronary artery revascularisation procedures following their ED chest pain visit, but no ICA. For these, we assumed that an angiogram was performed but not coded because revascularisation procedures cannot be performed without imaging details that show the location and extent of stenosis in the coronary arteries. Cardiologists and surgeons require this information prior to attempting any revascularisation procedure, whether it is percutaneous coronary intervention or coronary artery bypass surgery. It is possible that instead of ICA some patients may have had CCTA outside of the hospital sector (e.g., in private radiology clinics), and these would not be captured through the inpatient admissions data.

## 5. Conclusions

Only one in five ED presentations for chest pain are discharged from ED with a diagnosis of cardiovascular disease, with 10.4% diagnosed with ACS. Men and women are equally represented in ED chest pain visits, but have significantly different demographic and clinical characteristics. This may have influenced the frequency of use of ICA between men and women following ED discharge, but the timing was the same, with 84% of angiograms initiated in the first 7 days for men and women. The most common pathways to initiation of angiograms were direct admission to hospital with/without immediate procedure and discharge home with subsequent procedure within 90 days. Subsequent investigation of ED chest pain presentations will reveal cost effectiveness and outcomes for the various pathways to ICA, especially in patients with contiguous presentations to ED for chest pain.

## Figures and Tables

**Table 1:** Characteristics of patients aged 20 years or older with presenting complaint of chest pain to public tertiary hospital emergency departments from 1 Jan 2016 to 31 Mar 2017 (ED chest pain cohort). Percentages are based on total number of patients in the ED chest pain cohort (*n* = 16,974).

Characteristic	Count, *n* (% of Total Patients)	Patients with >1 ED Visit, *n* (%)
Number of ED visits	20,131	5115
Number of patients	16,974	1958 (11.5)
Sex ^†^ Males	8609 (50.7)	1013 (51.7)
Females	8364 (49.3)	945 (48.3)
Age, mean (SD) years	55.6 (19.1)	59.6 (19.0)
Age range (years)	20–103	20–101
Age group (years)		
20–44	5273 (31.1)	446 (22.8)
45–64	5815 (34.3)	669 (34.2)
65–74	2565 (15.1)	343 (17.5)
≥75	3321 (19.6)	500 (25.5)
Indigenous Australians	809 (4.8)	162 (8.3)
Triage code (of first ED presentation in study period)		
1. Resuscitation	129 (0.8)	10 (0.5)
2. Emergency	15,780 (93.0)	1882 (96.1)
3. Urgent	861 (5.1)	58 (3.0)
4. Semi-urgent	189 (1.1)	7 (0.4)
5. Non-urgent	15 (0.1)	1 (0.05)
Remoteness area ^‡^		
Major cities	15,767 (92.9)	1860 (95.0)
Inner regional	173 (1.0)	14 (0.7)
Outer regional	210 (1.2)	18 (0.9)
Remote	91 (0.5)	9 (0.5)
Very remote	69 (0.4)	6 (0.3)
Missing	664 (3.9)	51 (2.6)
Medical history (10 year lookback)		
Coronary heart disease	2447 (14.4)	491 (25.1)
Diabetes	2388 (14.1)	469 (24.0)
Hypertension	3646 (21.5)	735 (37.5)
Atrial fibrillation	1619 (9.5)	332 (17.0)
PCI or CABG	1393 (8.2)	309 (15.8)
Mean time (SD) from most recent PCI/CABG in past 10 years to ED presentation	1267 (1051) days 3.5 (2.9) years	1184 (1055) days 3.2 (2.9) years
Died within 30 days of ED discharge (in or out of hospital)	149 (0.9) ^¶^	9 (0.5)
Died within 90 days of ED discharge (in or out of hospital)	311 (1.8) ^¶^	40 (2.0)

ED = emergency department; SD = standard deviation; PCI, percutaneous coronary intervention (with/without stents); CABG, coronary artery bypass graft surgery; SD, standard deviation. ^†^ Sex was missing for one patient. ^‡^ Grouped as remoteness areas based on the Accessibility Remoteness Index of Australia (ARIA+) using Statistical Area Level 2 (SA2) from the 2011 census [14]. ^¶^ Based on first ED visit in study period for each patient.

**Table 2:** Frequency and timing of initial invasive coronary angiography within 90 days of the first ED presentation for each person in the ED Chest Pain Cohort (*n* = 16,974).

Covariate	Number of Initial Angiograms (row %) after the First ED Presentation ^†^			*p* Value	Total (col %)
	0–7 days	8–30 days	31–90 days		
Total	1300 (84.1)	118 (7.6)	128 (8.3)		1546
Troponin I test result				<0.0001 ^‡^	
No tests	39 (66.1)	7 (11.9)	13 (22.0)		59 (3.8)
All normal	392 (69.4)	81 (14.3)	92 (16.3)		565 (36.6)
Elevated ^§^	869 (94.2)	30 (3.2)	23 (2.5)		922 (59.6)
Sex				0.28 ^‡^	
Males	879 (84.2)	85 (8.1)	80 (7.7)		1044 (67.5)
Females	421 (83.9)	33 (6.6)	48 (9.6)		502 (32.5)
Age group (years)				0.09 ^‡^	
20–44	92 (86.0)	8 (7.5)	7 (6.5)		107 (6.9)
45–64	576 (84.0)	59 (8.6)	51 (7.4)		686 (44.4)
65–74	342 (83.6)	21 (5.1)	46 (11.3)		409 (26.5)
≥75	290 (84.3)	30 (8.7)	24 (7.0)		344 (22.2)
Remoteness area ^¶^				0.45 ^£^	
Major cities	1165 (84.0)	106 (7.7)	115 (8.3)		1386 (89.6)
Regional	51 (76.1)	7 (10.5)	9 (13.4)		67 (4.3)
Remote, very remote	32 (91.4)	2 (5.7)	1 (2.9)		35 (2.3)
Missing	52 (89.6)	3 (5.2)	3 (5.2)		58 (3.8)
ED discharge diagnosis				<0.0001 ^‡^	
MI	655 (97.3)	13 (1.9)	5 (0.7)		673 (43.5)
Unstable angina	224 (85.8)	21 (8.0)	16 (6.1)		261 (16.9)
Other angina	39 (65.0)	11 (18.3)	10 (16.7)		60 (3.9)
Other CHD	7 (87.5)	1 (12.5)	0		8 (0.5)
Other CVD	76 (76.8)	10 (10.1)	13 (13.1)		99 (6.4)
Chest pain	237 (67.9)	46 (13.2)	66 (18.9)		349 (22.6)
Non-CVD	62 (64.6)	16 (16.7)	18 (18.8)		96 (6.2)

ED, emergency department; MI, myocardial infarction; CHD, coronary heart disease; CVD, cardiovascular disease. ^†^ If admissions within 90 days of first ED presentation had a coronary artery revascularisation procedure (percutaneous coronary intervention or coronary artery bypass graft surgery), but no procedure codes for coronary angiography, then we assumed angiography was performed and not coded. ^‡^ 2-sided Pearson Chi-squared test. ^§^ Troponin test results were either all elevated above the normal cut off for the laboratory or in a rising or falling pattern with at least 1 test result elevated. ^¶^ Grouped as remoteness areas based on the Accessibility Remoteness Index of Australia (ARIA+) using Statistical Area Level 2 (SA2) from the 2011 census [14]. ^£^ Fisher’s exact test.

**Table 3:** Multivariate logistic regression model to estimate odds of initiating invasive coronary angiography within 90 days of first ED chest pain visit from 1 Jan 2016 to 31 Mar 2017 (*n* = 16,974).

Variable	Adjusted Odds Ratio (95% CI)	*p* Value ^‡^
Age	0.99 (0.99, 1.00)	0.0006
Male sex (reference level: females)	1.89 (1.65, 2.18)	<0.0001
Remoteness area (reference level: major cities)		<0.0001
Regional (inner/outer)	2.11 (1.49, 2.99)	
Remote, very remote	2.53 (1.54, 4.16)	
Missing	1.18 (0.83, 1.68)	
ED discharge diagnosis (reference level: non-CVD)		<0.0001
Myocardial infarction	32.75 (24.95, 42.97)	
Unstable angina	18.47 (14.16, 24.11)	
Other angina	8.93 (6.21, 12.84)	
Other CHD	14.48 (5.36, 39.13)	
Other CVD	2.35 (1.75, 3.17)	
Chest pain	2.02 (1.60, 2.55)	
Troponin level (reference: troponin test not done)		<0.0001
All tests normal	3.34 (2.52, 4.44)	
Elevated ^†^	14.37 (10.55, 19.58)	

Model C statistic = 0.89. ED, emergency department; 95% CI, 95% confidence, interval; CHD, coronary heart disease; CVD, cardiovascular disease. History of CHD, diabetes, hypertension, atrial fibrillation and coronary artery revascularisation procedures were not included in the model due to a high correlation of CHD history with ED discharge diagnosis, and very little impact of these variables on the model c statistic. ^†^ Troponin test results were either all elevated above the normal cut off for the laboratory or in a rising or falling pattern with at least 1 test result elevated. ^‡^ Type 3 Wald Chi-squared *p* value.

## Supplementary Materials

The following are available online at https://www.mdpi.com/1660-4601/17/24/9502/s1, Table S1: Counts (row%) of Emergency Department (ED) discharge diagnosis compared with corresponding principal discharge diagnosis of the inpatient admission for patients in the ED chest cohort admitted to hospital directly from the ED (*n* = 8469 visits). Table S2: Number and type of cardiac biomarker test identified within 1 h prior to and 6 h after the emergency department (ED) presentation time in the ED chest pain cohort. Table S3: Troponin I test results by emergency department (ED) discharge diagnosis in the ED chest pain cohort. Table S4: Admissions for initial invasive coronary angiogram within 90 days and after 90 days from the first ED presentation for each person in the ED Chest Pain Cohort (*n* = 16,974). Table S5: Patients with a normal troponin level who had invasive coronary angiography within 90 days of their first ED chest pain visit from 1 Jan 2016 to 31 Mar 2017 (*n* = 565). Table S6: Gender differences in demographic and clinical characteristics in the ED chest pain cohort (counts are person-based *n* = 16,974).

## Data Sharing and Accessibility

We will consider requests for data sharing on an individual basis, with the aim to share data whenever possible for appropriate research purposes. However, this research project uses data obtained from a third-party source under strict privacy and confidentiality agreements from the Western Australian Department of Health databases, which are governed by their ethics committee and data custodians. The data were provided after approval was granted from their standard application processes for access to the linked datasets. Therefore, any requests to share these data with other researchers will be subject to formal approval from the third-party ethics committees and data custodians. Researchers interested in these data should contact the Client Services Team at the Data Linkage Branch of the Western Australian Department of Health (www.datalinkage-wa.org.au/contact-us).

## References

[1] National Center for Health Statistics National Hospital Ambulatory Medical Care Survey: 2017 Emergency Department Summary Tables. https://www.cdc.gov/nchs/data/nhamcs/web_tables/2017_ed_web_tables-508.pdf.

[2] Emergency Medicine Foundation New Research: Diagnosing Chest Pain Quickly. https://emergencyfoundation.org.au/2017/09/04/new-research-diagnosing-chest-pain-quickly/.

[3] Australian Institute of Health and Welfare (2018). Emergency Department Care 2017–18: Australian Hospital Statistics.

[4] Chew D.P., Scott I.A., Cullen L., French J.K., Briffa T.G., Tideman P.A., Woodruffe S., Kerr A., Branagan M., Aylward P.E. (2016). National Heart Foundation of Australia & Cardiac Society of Australia and New Zealand: Australian clinical guidelines for the management of acute coronary syndromes 2016. Heart Lung Circ..

[5] Cullen L., Greenslade J., Merollini K., Graves N., Hammett C.J., Hawkins T., Than M.P., Brown A.F., Huang C.B., Panahi S.E. (2015). Cost and outcomes of assessing patients with chest pain in an Australian emergency department. Med. J. Aust..

[6] Andruchow J.E., Kavsak P.A., McRae A.D. (2018). Contemporary emergency department management of patients with chest pain: A concise review and guide for the high-sensitivity troponin era. Can. J. Cardiol..

[7] Australian Institute of Health and Welfare (2019). Disease Expenditure in Australia 2015–16.

[8] Nedkoff L., Goldacre R., Greenland M., Goldacre M.J., Lopez D., Hall N., Knuiman M., Hobbs M., Sanfilippo F.M., Wright F.L. (2019). Comparative trends in coronary heart disease subgroup hospitalisation rates in England and Australia. Heart.

[9] Holman C.D.A.J., Bass J.A., Rosman D.L., Smith M.B., Semmens J.B., Glasson E.J., Brook E.L., Trutwein B., Rouse I.L., Watson C.R. (2008). A decade of data linkage in Western Australia: Strategic design, applications and benefits of the WA data linkage system. Aust. Health Rev..

[10] Thygesen K., Alpert J.S., Jaffe A.S., Chaitman B.R., Bax J.J., Morrow D.A., White H.D. (2018). Fourth Universal Definition of Myocardial Infarction (2018). Circulation.

[11] Australian Consortium for Classification Development (2015). International Statistical Classification of Diseases and Related Health Problems, Tenth Revision, Australian Modification.

[12] Brenna C.T.A., Afgani F.J., Hanneman K., Levitan D., Udell J.A., Sacha Bhatia R., Harvey P.J., Nguyen E.T. (2020). Chest pain investigation in patients at low or intermediate risk: What is the best first-line test to rule out coronary artery disease?. Can. Fam. Phys..

[13] Fordyce C.B., Newby D.E., Douglas P.S. (2016). Diagnostic strategies for the evaluation of chest pain: Clinical implications from SCOT-HEART and PROMISE. J. Am. Coll. Cardiol..

[14] Australian Bureau of Statistics The Australian Statistical Geography Standard (ASGS) Remoteness Structure. https://www.abs.gov.au/websitedbs/D3310114.nsf/home/remoteness+structure.

[15] Bhuiya F.A., Pitts S.R., McCaig L.F. (2010). Emergency Department Visits for Chest Pain and Abdominal Pain: United States, 1999–2008.

[16] Greenslade J.H., Carlton E.W., Van Hise C., Cho E., Hawkins T., Parsonage W.A., Tate J., Ungerer J., Cullen L. (2018). Diagnostic accuracy of a new high-sensitivity troponin I assay and five accelerated diagnostic pathways for ruling out acute myocardial infarction and acute coronary syndrome. Ann. Emerg. Med..

[17] Diamond G.A., Kaul S. (2010). How would the reverend Bayes interpret high-sensitivity troponin?. Circulation.

[18] Chew D.P., MacIsaac A.I., Lefkovits J., Harper R.W., Slawomirski L., Braddock D., Horsfall M.J., Buchan H.A., Ellis C.J., Brieger D.B. (2016). Variation in coronary angiography rates in Australia: Correlations with socio-demographic, health service and disease burden indices. Med. J. Aust..

[19] Haider A., Bengs S., Luu J., Osto E., Siller-Matula J.M., Muka T., Gebhard C. (2020). Sex and gender in cardiovascular medicine: Presentation and outcomes of acute coronary syndrome. Eur. Heart J..

