# Supplementary material for: Invasive Coronary Angiography after Chest Pain Presentations to Emergency Departments
Source: Int J Environ Res Public Health. 2020 Dec 18;17(24):9502. doi: 10.3390/ijerph17249502 (PMC7766965; doi:10.3390/ijerph17249502)
Supplement: Supplementary file 1 [file ijerph-17-09502-s001.pdf]

Supplementary File

Invasive coronary angiography after chest pain presentations to emergency departments

Sanfilippo FM, Hillis G, Rankin JM, Latchem D, Schultz C, Yong Y, Li I, Briffa TG

Table S1: Counts (row%) of Emergency Department (ED) discharge diagnosis compared with corresponding principal discharge diagnosis of the inpatient admission for patients in the ED chest cohort admitted to hospital directly from the ED (n=8469 visits).

ED discharge diagnosis	Principal discharge diagnosis of corresponding inpatient admission						Total
	MI	UA	Other angina	Other CHD	Chest pain	Non-CHD	
MI	690 (68.5)	48 (4.8)	18 (1.8)	27 (2.7)	72 (7.2)	152 (15.1)	**1007**
UA	95 (12.9)	241 (32.6)	79 (10.7)	27 (3.6)	184 (24.9)	113 (15.3)	**739**
other angina	20 (10.4)	27 (14.0)	51 (26.4)	5 (2.6)	65 (33.7)	25 (12.9)	**193**
other CHD	<5	<5	<5	<5	<5	5 (31.2)	**16**
chest pain	154 (4.9)	134 (4.2)	193 (6.1)	22 (0.7)	1793 (56.6)	874 (27.6)	**3170**
non-CHD	86 (2.6)	30 (0.9)	42 (1.3)	11 (0.3)	324 (9.7)	2851 (85.3)	**3344**
**Total**	**1049**	**481**	**385**	**94**	**2440**	**4020**	**8469**

Counts represent number of visits.
MI = myocardial infarction; UA = unstable angina; CHD = coronary heart disease.
ICD-10-AM codes: MI I21; UA I20.0; other angina I20.1-I20.9; other CHD I22-I25; chest pain R07.1 to R07.4; non-CHD = all other ICD-10-AM codes.

The green cells indicate the number of visits with the same discharge diagnosis between ED and the corresponding inpatient admission. Diagnoses to the left of the green cells indicate an upgrading of discharge diagnosis once the patient was admitted as an inpatient (ie more serious diagnosis than the ED discharge diagnosis). Diagnoses to the right of the green cells indicate a downgraded diagnosis after the patient was admitted as an inpatient (ie less serious diagnosis than the ED discharge diagnosis).

Table S2: Number and type of cardiac biomarker test identified within 1 hour prior to and 6 hours after the emergency department (ED) presentation time in the ED chest pain cohort.

Type of test	Number of ED visits (%) with biomarker tests				
	No tests recorded †	1 test	2 tests	3 tests	4 tests
Troponin I ‡	5589 (27.8)	7026 (34.9)	7246 (36.0)	267 (1.3)	<5
CK §	19,764 (98.2)	349 (1.7)	18 (0.1)	-	-

† No troponin tests recorded in the pathology dataset within 1 hour prior to or 6 hours after the ED presentation time.
‡ Includes troponin I normal sensitivity (n=1) and high sensitivity (n=14,541). Number of patients with no troponin tests in any of their ED visits is 4570; number of patients with a total of 1 troponin test from all their ED visits is 5468; and the number of patients with a total of ≥2 troponin tests from all their ED visits is 6936.
§ CK = creatine kinase (no CK-MB isoenzyme tests were done) and is most likely ordered to look for skeletal muscle damage not as a cardiac biomarker.

Our data did not include ED discharge date and time, so some of the troponin tests may have been done during inpatient admission (we only identified tests completed within 1 hour prior to and 6 hours after the ED presentation time).

Table S3: Troponin I test results by emergency department (ED) discharge diagnosis in the ED chest pain cohort.

ED discharge diagnosis	Number of visits (row %) for each troponin I test result				Total
	No tests †	All normal	All elevated	≥1 elevated with rise/fall pattern ‡	
MI	18 (1.7)	95 (8.8)	894 (82.4)	78 (7.2)	**1085**
UA	42 (4.1)	723 (71.1)	220 (21.6)	32 (3.2)	**1017**
other angina	25 (6.0)	320 (76.6)	54 (12.9)	19 (4.6)	**418**
other CHD	<5	8 (36.4)	13 (59.1)	0	**22**
other CVD	605 (32.0)	873 (46.2)	370 (19.6)	41 (2.2)	**1889**
chest pain	2272 (23.6)	6745 (70.0)	489 (5.1)	132 (1.4)	**9638**
non-CVD	2626 (43.3)	3013 (49.7)	369 (6.1)	54 (0.9)	**6062**
**Total, n (%)**	**5589 (27.8)**	**11,777 (58.5)**	**2409 (12.0)**	**356 (1.8)**	**20,131**

† No troponin test records were present in the pathology dataset for 5589 ED visits in the ED chest pain cohort.
‡ Troponin test results in a rise or fall pattern with at least 1 test result elevated above the normal cutoff for the laboratory. Rounding may mean that percentages do not equate to 100.

MI = myocardial infarction, UA = unstable angina, CHD = coronary heart disease, CVD = cardiovascular disease. ICD-10-AM codes: MI I21; UA I20.0; other angina I20.1-I20.9; chest pain R07.1 to R07.4; other CHD I22-I25; other CVD = all ICD-10-AM chapter I codes other than I20-I25; non-CVD = all ICD-10-AM codes other than I00-I99 and R07.1 to R07.4.

Table S4: Admissions for initial invasive coronary angiogram within 90 days and after 90 days from the first ED presentation for each person in the ED Chest Pain Cohort (n=16,974).

Covariate	Number of initial angiograms (row %) after the first ED presentation †		p value	Total (col %)
	within 0-90 days	>90 days ‡		
**Total count**	1546 (88.1)	209 (11.9)		1755
**Troponin I test result**			<0.0001 §	
No tests	59 (69.4)	26 (30.6)		85 (4.8)
All normal	565 (79.2)	148 (20.8)		713 (40.6)
Elevated ¶	922 (96.3)	35 (3.7)		957 (54.6)
**Sex**			0.026 §	
Males	1044 (89.3)	125 (10.7)		1169 (66.6)
Females	502 (85.7)	84 (14.3)		586 (33.4)
**Age group (years)**			0.73 §	
20-44	107 (86.3)	17 (13.7)		124 (7.0)
45-64	686 (87.7)	96 (12.3)		782 (44.6)
65-74	409 (88.0)	56 (12.0)		465 (26.5)
≥ 75	344 (89.6)	40 (10.4)		384 (21.9)
**Remoteness area £**			0.22 Ψ	
Major cities	1386 (87.7)	195 (12.3)		1581 (90.1)
Regional	67 (88.2)	9 (11.8)		76 (4.3)
Remote, very remote	35 (94.6)	<5		37 (2.1)
Missing	58 (95.1)	<5		61 (3.5)
**ED discharge diagnosis**			<0.0001 §	
MI	673 (98.7)	9 (1.3)		682 (38.9)
Unstable angina	261 (90.9)	26 (9.1)		287 (16.3)
Other angina	60 (88.2)	8 (11.8)		68 (3.9)
Other CHD	8 (80.0)	<5		10 (0.6)
Other CVD	99 (81.8)	22 (18.2)		121 (6.9)
Chest pain	349 (79.0)	93 (21.0)		442 (25.1)
Non-CVD	96 (66.2)	49 (33.8)		145 (8.3)

† If admissions within 90 days of first ED presentation had a coronary artery revascularisation procedure (percutaneous coronary intervention or coronary artery bypass graft surgery), but no procedure codes for coronary angiography, then we assumed an angiography was performed and not coded.
‡ to 30 June 2017.
§ 2-sided Pearson chi-squared test.
¶ Troponin test results were either all elevated above the normal cutoff for the laboratory or in a rising or falling pattern with at least 1 test result elevated.
£ Grouped as Remoteness Areas based on ARIA+ (Accessibility Remoteness Index of Australia) using Statistical Area Level 2 (SA2) from the 2011 census.[1]
Ψ Fisher's exact test.

Table S5: Patients with a normal troponin level who had invasive coronary angiography within 90 days of their first ED chest pain visit during 1 Jan 2016 to 31 Mar 2017 (n=565).

**Characteristic**		**Count, n (%)**
**Sex**	males	384 (68.0)
	females	181 (32.0)
**Age, mean (SD)**		63.2 (12.0) years
**Age group (years)**		
	20-44	40 (7.1)
	45-64	249 (44.1)
	65-74	169 (29.9)
	≥ 75	107 (18.9)
**Remoteness area** †		
	Major cities	504 (89.2)
	Inner regional	11 (2.0)
	Outer regional	19 (3.4)
	Remote	8 (1.4)
	Very remote	7 (1.2)
	Missing	16 (2.8)
**ED discharge diagnosis**		
	MI	67 (11.9)
	Unstable angina	177 (31.3)
	Other angina	41 (7.2)
	Other coronary heart disease	<5
	Other cardiovascular disease	34 (6.0)
	Chest pain	197 (34.9)
	Non-cardiovascular disease	45 (8.0)
**Medical history (10-year lookback)**		
	Myocardial infarction	94 (16.6)
	Coronary heart disease ‡	265 (46.9)
	Diabetes	136 (24.1)
	Hypertension	171 (30.3)
	Atrial fibrillation	38 (6.7)
	PCI or CABG	111 (19.6)
	Mean time (SD) from most recent PCI/CABG in past 10 years to ED presentation	1361 (1016) days<br>3.7 (2.8) years

ED, emergency department; PCI, percutaneous coronary intervention (with/without stents); CABG, coronary artery bypass graft surgery; SD, standard deviation.
† grouped as Remoteness Areas based on the Accessibility Remoteness Index of Australia (ARIA+) using Statistical Area Level 2 (SA2) from the 2011 census.[1]
‡ Coronary heart disease includes the myocardial infarction count.

Table S6: Gender differences in demographic and clinical characteristics in the ED chest pain cohort (counts are person-based n=16,974).

Covariate	Males (n=8609)	Females (n=8364)	p value †
**Age group (years)**			<0.0001
20-44	2695 (31.3)	2578 (30.8)	
45-64	3037 (35.3)	2777 (33.2)	
65-74	1336 (15.5)	1229 (14.7)	
≥ 75	1541 (17.9)	1780 (21.3)	
**Remoteness area** ‡			<0.0001
Major cities	7915 (91.9)	7851 (93.9)	
Regional (inner/outer)	212 (2.5)	171 (2.0)	
Remote, very remote	85 (1.0)	75 (0.9)	
Missing	397 (4.6)	267 (3.2)	
**ED discharge diagnosis** §			<0.0001
MI	608 (7.1)	351 (4.2)	
Unstable angina	449 (5.2)	286 (3.4)	
Other angina	174 (2.0)	136 (1.6)	
Other coronary heart disease	11 (0.1)	7 (0.1)	
Other cardiovascular disease	825 (9.6)	693 (8.3)	
Chest pain	4053 (47.1)	4245 (50.8)	
Non-cardiovascular disease	2489 (28.9)	2646 (31.6)	
**Comorbidities (10-year history)**			
coronary heart disease	1431 (16.6)	1016 (12.2)	<0.0001
diabetes	1296 (15.1)	1092 (13.1)	0.0002
hypertension	1944 (22.6)	1702 (20.4)	0.0004
atrial fibrillation	894 (10.4)	725 (8.7)	0.0001
**Troponin I test result** §			<0.0001
No tests	2071 (24.1)	2728 (32.6)	
All normal	5262 (61.1)	4675 (55.9)	
Elevated ¶	1276 (14.8)	961 (11.5)	
**Initial angiogram (any time from ED visit)** §			<0.0001
No	7440 (86.4)	7778 (93.0)	
Yes	1169 (13.6)	586 (7.0)	
**Initial angiogram within 90 days of ED visit** §			<0.0001
No	7565 (87.7)	7862 (93.9)	
Yes	1044 (12.3)	502 (6.1)	

1 person had value for sex missing.
ED = emergency department; MI = myocardial infarction; CVD = cardiovascular disease.
† 2-sided Pearson chi-squared test.
‡ Grouped as Remoteness Areas based on ARIA+ (Accessibility Remoteness Index of Australia) using Statistical Area Level 2 (SA2) from the 2011 census.[1]
§ For first ED chest pain presentation in study period.
¶ Troponin test results were either all elevated above the normal cut-off for the laboratory or in a rising or falling pattern with at least 1 test result elevated.

References

1. Australian Bureau of Statistics. The Australian Statistical Geography Standard (ASGS) remoteness structure. 2020 [cited: 10 July 2020]. https://www.abs.gov.au/websitedbs/D3310114.nsf/home/remoteness+structure
